# The Amphoteric and Hydrophilic Properties of Cartilage Surface in Mammalian Joints: Interfacial Tension and Molecular Dynamics Simulation Studies

**DOI:** 10.3390/molecules24122248

**Published:** 2019-06-16

**Authors:** Katarzyna Janicka, Piotr Beldowski, Tomasz Majewski, Wieslaw Urbaniak, Aneta D. Petelska

**Affiliations:** 1 Institute of Chemistry, University of Bialystok, Ciolkowskiego 1K, 15-425 Bialystok, Poland; k.janicka@uwb.edu.pl; 2 Institute of Mathematics and Physics, University of Technology and Life Sciences, Kaliskiego 7, 85-796 Bydgoszcz, Poland; piotr.beldowski@utp.edu.pl; 3 Institute for Multiscale Simulation, Cluster of Excellence ”Engineering of Advanced Materials”, Friedrich-Alexander-Universität Erlangen-Nürnberg, Cauerstrasse 3, 91058 Erlangen, Germany; 4 Institute of Armament Technology, Faculty of Mechatronics and Aerospace, Military University of Technology, Gen. Sylwestra Kaliskiego 2, 00-908 Warsaw, Poland; tomasz.majewski@wat.edu.pl; 5 Faculty of Mathematics, Physics and Technical Sciences, Kazimierz Wielki University, Chodkiewicza 30, 85-867 Bydgoszcz, Poland; wurban@ukw.edu.pl

**Keywords:** interfacial tension, molecular dynamics simulation, phospholipids bilayers, amphoteric articular cartilage

## Abstract

In this paper, we explain the amphoteric character of the cartilage surface by studying a lipid bilayer model built from phospholipids. We examined the interfacial tension values and molecular dynamics simulation in solutions of varying pH. The effects of negative and positive charge density (or fixed charges) on the (cartilage/cartilage) friction coefficient were investigated. In physiological (or synovial) fluid, after the isoelectric point (pI), the curve of interfacial tension decreases rapidly as it reaches pH 7.4 and then approaches a constant value at higher pH. It was shown that the curve of the interfacial tension curve exhibits a maximum value at the isoelectric point with a Gaussian shape feature. The phospholipid bilayers facilitate an almost frictionless contact in the joint. Moreover, the slippage of the bilayer and the short-range repulsion between the surfaces of the negatively charged cartilage surfaces are the main determinants of the low frictional properties of the joint.

## 1. Introduction

The paper presents the application of a lamellar-repulsion model of the charge density of the interaction between amphoteric surfaces to depict the friction of the sliding surfaces made of cartilage [1,2,3,4]. Following this model, low friction can be observed while repulsive articular cartilage surfaces slide against one another with a hydration water layer in between, which is operating as a lubricant. Due to this, the friction may be lowered by the bilayer lamellar slippage, a repulsion over short distances, and the water in the synovial fluid. Therefore, the friction caused by the sliding of two surfaces charged with the same sign was studied [5,6,7,8,9]. Oloyede et al. showed the strong dependence of the friction from both the cartilage surface charge density and the surface wettability by using contact angle and friction coefficient measurements using the lamellar repulsion model [10]. The natural cartilage of animal joints has very low friction for the sliding velocity; a few centimeters per second under a load of 18 MPa [11].

**Figure 1:**
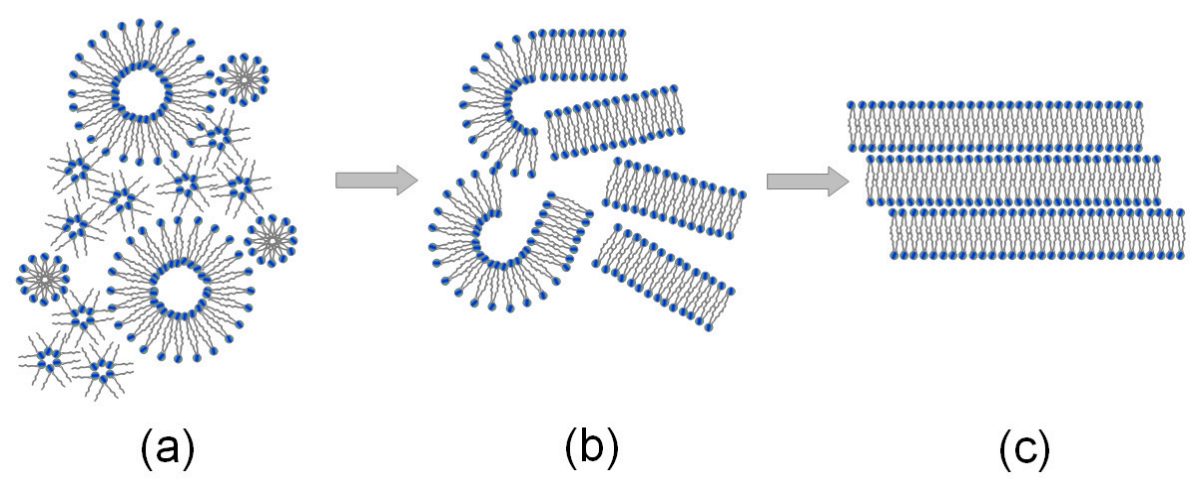
Various phospholipid formations: (**a**) liposomal and hexagonal structures in the synovial fluid, (**b**) lamellar phases in the synovial fluid under load, and (**c**) phospholipid bilayers on the cartilage surface under load.

Figure 1 shows the self-organization of lipid liposomes and hexagonal structures in synovial fluid, the formation of the phospholipids’ lamellar phases under load, and finally, the lamellar slippage of the phospholipid bilayers on the surface of the cartilage under load. The negatively charged surface of the cartilage with synovial fluid at pH 7.4 is supported under load by the lamellar slippage of bilayers, thus resulting in low friction. It was experimentally proven that phospholipids, which are in the synovial fluid or form the articular cartilage surface, play an important role as a lubricant, but in the case of the deformation of the phospholipids’ bilayer surfaces, the lubrication of the joints deteriorates [12,13,14].

As shown by Petelska et al. [9] for different cartilages, different values of the friction coefficient have been observed. The curves presented in their paper [9] should be related to the amphoteric character and charge density. The results show that the friction coefficient is highly reliant on the electrostatic interaction between the two cartilage surfaces. The observed low friction of the two sliding cartilage sides that have the same charge is associated with the electrostatic repulsion of a charged cartilage/cartilage pair.

Other authors have presented more information about new materials and modified lubricin structures for low friction [15] such as surface-grafted polymer brushes [16], lubricin-like synthetic copolymers [17], polymer-peptide-based surface coatings to bind HA (which is part of the synovial fluid) non-covalently to surfaces [18], self-assembled monolayer functionalized model substrates with hydroxyl- or methyl-terminating groups to study the effect of surface chemistry on lubricin and HA adsorption [19] as well as the copolymer polyacrylic acid-co-polyethylene glycol to simulate lubricin and its interaction with fibronectin on mica surfaces [20].

In this paper, we studied the relationship between pH by the interfacial tension of spherical lipid bilayers built of phospholipids and molecular dynamic simulation. The amphoteric cartilage surface change charge in a pH range of 1–9, from positive (–NH_3_^+^), neutral, which is the isoelectric point (pI) (the pH values at which a particular molecule carries no net electrical charge), and negative (–PO_4_^−^). The friction coefficient of the (cartilage/cartilage) pair was measured in the following cases: both cartilages were positively charged (–NH_3_^+^)/(–NH_3_^+^), then negatively charged (–PO_4_^−^)/(–PO_4_^−^), and finally neutral. Studying the friction in (cartilage/cartilage) pairs of bovine origin over the pH of buffer solutions is fascinating to observe the electrostatic mechanism of joint lubrication.

We also used molecular dynamics simulations and the approach of a small world network to study the dynamic couplings using a distance map applied to oxygen atoms in the DPPC (phosphatidylcholine) and DPPE (phosphatidylethanolamine) chains and water. DPPE creates ~150% more water bridges than the DPPC bilayer. However, compared to DPPC, these bridges do not last as long.

## 2. Results and Discussion

### 2.1. The Interfacial Tension of the Phospholipid Membrane

**Figure 2:**
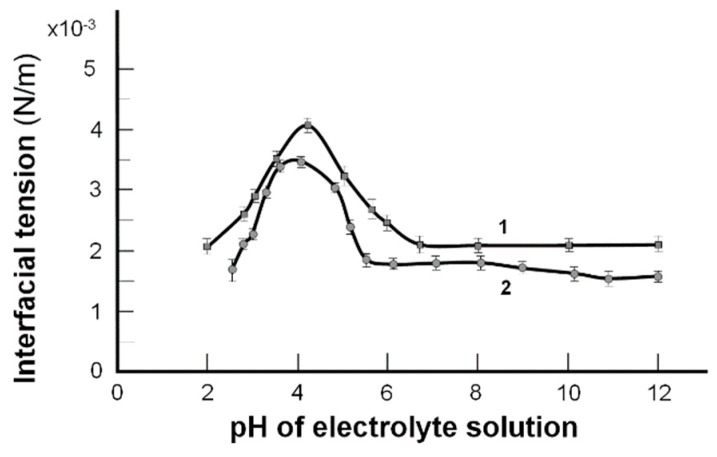
The interfacial tension values of the model spherical bilayers formed from phosphatidylethanolamine (1) and phosphatidylcholine (2) vs. the pH of the electrolyte solution (the experimental values are indicated by point, and the theoretical values are indicated by the curves).

The graphs (Figure 2) show that the maximal values of interfacial tension occurred at the isoelectric point. The trends of these curves have been well characterized by a simplified description based on the Gibbs isotherm, but only near the isoelectric point [21].

Using the exact definition of surface excess in the Gibbs equation allows us to explain the course of experimental curves across the entire pH range [22,23,24,25]. The changes of the bilayer interfacial tension values on the pH solution can be described by the acid–base equilibria presented below [22]:

(1) $$\begin{array}{c} PO_{4}^{-}+H^{+}\to PO_{4}^{-}-H^{+}\\ \;(X^{-})\;(\mathrm{XH}) \end{array}$$

(2) $$\begin{array}{c} NH_{3}^{+}+OH^{-}\to NH_{3}^{+}-OH^{-}\\ \left(Y^{+})\;(\mathrm{YOH}\right) \end{array}$$

(3) $$\gamma =\gamma_{X^{-}}^{0}(\frac{1}{1+K_{X}a_{H^{+}}})+\gamma_{\mathrm{XH}}^{0}(\frac{K_{X}a_{H^{+}}}{1+K_{X}a_{H^{+}}})+\gamma_{Y^{+}}^{0}(\frac{1}{1+K_{Y}a_{{\mathrm{OH}}^{-}}})+\gamma_{\mathrm{YOH}}^{0}(\frac{K_{Y}a_{{\mathrm{OH}}^{-}}}{1+K_{Y}a_{{\mathrm{OH}}^{-}}})$$

where $K_{X}$ and $K_{Y}$ are the acid and base equilibrium constants, respectively; γ [N/m] is the measured interfacial tension of the bilayer; $\gamma_{X^{-}}^{0},\;\;\gamma_{XH}^{0},\;\;\gamma_{Y^{+}}^{0},\;\;\gamma_{YOH}^{0}$ [N/m] are specific interfacial tension values of the bilayer components, respectively; $a_{H^{+}}$, $a_{{\mathrm{OH}}^{-}}$ are H^+^ and OH^−^ ions concentration, respectively.

Equation (3) shows the dependence of the interfacial tension of the lipid bilayer on the pH of the electrolyte solution. Experimental values in Figure 2 and the theoretical ones obtained from Equation (3) shown above are indicated by points and lines, respectively. The isoelectric point, pI, has a pH of ~4 [23,24] when the phospholipid molecule has a net charge of zero H_2_N (CH_2_)_n_ PO_4_H-R_1_R_2_ ⇆ H_3_N^+^ (CH_2_)_n_ PO_4_^−^-R_1_R_2_. Changes in interfacial tension correspond to the amine conversion (-NH_3_ + → –NH_2_) at low pH, after the pI (DPPC, pH 4.14; DPPE, pH 4.20, and phosphate (–PO_4_H → –PO_4_^−^)) transition at a higher pH range from 4.14 to 6.5.

In Figure 2, the isoelectric point, pI, with a pH at which a phospholipid molecule (e.g., phosphatidylethanolamine) carries no net electrical charge, is expressed as follows:

pI (pH 4.20); H_2_N (CH_2_)_n_ PO_4_H–R_1_R_2_ ⇆ H_3_N^+^(CH_2_)_n_ PO_4_^−^–R_1_R_2_
pH 1 to 4.20 (–NH_3_^+^ → –NH_2_) (pH 4.20 to 6.5) (–PO_4_H → –PO_4_^−^)
(Left side of the curve) (Right side of the curve)

The acid and base constants of the lipid membranes were determined by titration [21]. However, it was difficult to determine these values as the phospholipid is insoluble in water. For this reason, the required values were determined using liposomes. For the calculations, it was assumed that only the lipid molecules present in the outer layers of the liposomes were involved in the determination of the acid–base constants. Therefore, the concentration of lipid used in the equations was half as much as in the solution. The acid–base constants of the lipid membranes were determined by titration of the previously obtained liposomes with HCl and NaOH solutions. After calculating the dissociation constants of the membrane, the isoelectric point can be calculated using the equations presented in [21]. The acid–base equilibria and the isoelectric point values for the lipid membrane built from DPPS, DPPC, and DPPE obtained in this way are presented in Table 1. The acid equilibrium value for DPPC and DPPE was almost the same and was equal to 10^2.58^ [21,24].

Table 1 presents the physicochemical parameters for the examined phospholipids: interfacial tension values at pI, the association constant, and the interfacial tension energy values for lipid membranes built from DPPS, DPPC, and DPPE.

It is also an interesting relationship that with the decrease of the diameter of the hydrophilic lipid head, the interfacial tension value of the isoelectric point of the lipid membrane increases.

With a giant hydrophilic head, the isoelectric point appears at a lower pH. Interestingly, the value of the interfacial tension of the isoelectric point increases with a reduction in the diameter of the hydrophilic lipid head [25]. It has been shown that the size of the hydrophilic head influences the values of interfacial tension and, consequently, the properties of the cell membrane. Unlike other phospholipids, e.g., DPPC or DPPS, the DPPE has a small hydrophilic head. The calculated interfacial tension values are, respectively, 4.0 × 10^−3^ N/m for DPPE, 3.5 × 10^−3^ N/m for DPPC, and 2.93 × 10^−3^ N/m DPPS (large hydrophilic head) [24].

Moreover, the surface energies determined in the pIs for DPPC and DPPE—related to 1 mole of the substance—were similar and amounted to 1800 J/mol [25].

**Figure 3:**
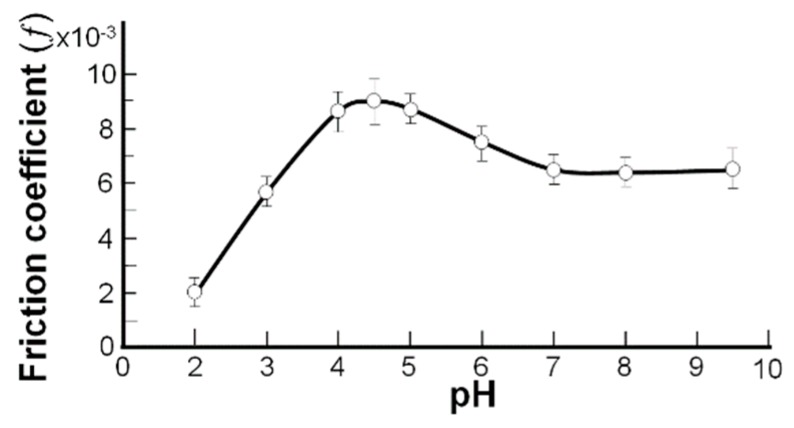
The friction coefficient of the bovine surface for pH 2.0–9.5 at a 15 N load and a slip speed of 1 mm/s during a 300 s run for each pH buffer solution.

The interfacial tension of the spherical lipid bilayers of the phospholipidic membrane vs. pH as determined by the interfacial tension measurements method supports the proposition of the amphoteric property of the phospholipidic cartilage surface (see Figure 3). A detailed description of the friction coefficient measurements has been presented in a previous paper [26]. The amphoteric molecules of phospholipids contain the functional –NH_2_ and –PO_4_H groups and are under the influence of the pH of the solution. While increasing the solution’s pH from 5.0 to 8.0 (after the isoelectric point), the –PO_4_H group begins to drop its proton (–PO_4_H + OH^−^ → –PO_4_^−^ + H_2_O), so the surface is a negatively charged surface, and the interfacial tension decreases.

A relationship between the interfacial tension of the model membrane (Figure 2) and the friction coefficients in a bovine cartilage pair vs. the pH of the buffer solutions is demonstrated in Figure 3. As pH is varied, the interfacial tension of the spherical bilayer’s membrane is formed by phosphatidylethanolamine (PE) (Curve 1, Figure 2) and phosphatidylcholine (Curve 2, Figure 2), and the friction coefficient (Figure 3) in a bovine cartilage pair reaches the maximum on the curves, which is remarkably similar amphoteric behavior occurring to that characterizing the pI.

Nature has equipped cartilage with synovial fluid in the form of an efficient amphoteric phospholipidic buffer mixture. The actual concentrations of [–PO_4_^−^] and [–PO_4_H] influence the effectiveness (or buffer capacity, β) expressed by the molar quantity of [OH^−^] added, and Δ*H^+^* is the molar amount of the added (or removed) protons to change the pH, so ΔpH results in Equation (4) [27].

ΔH^+^/ΔpH = β. (4)

The relationship between the buffer capacity (β) and the ratio of the components is represented by the Figure 2 curves (spherical lipid bilayers) and Figure 3 curves (cartilage) in the physiological range of 7.4 ± 0.7. Gradients of the tangents to the curves belonged to the interval (6.5, 9.5) and were inversely proportional to the buffer capacity (β = Δ (E_int tension_)/ΔpH) in Figure 2 and β = Δ (*f*)/ΔpH).

Below the isoelectric point of the cartilage surface and the bilayer’s model membrane, the surface was a positively charged (–NH_3_^+^) curve, with a gradual change in friction and interfacial tension as the pH shifted toward the pI. After passing through the pI, the surface charge gradually changed from positive (–NH_3_^+^) to negative (–PO_4_), while the surface friction changed from an attractive to a repulsive state. A similar trend was observed by Marti and co-workers [28] using an amphoteric material, SiO_2_. Relatively small slops in the tails of the curves in Figure 2 and Figure 3 were a good reason to refer to these portions as buffer regions with a very high buffer capacity (β) imparted by the phospholipid, ΔH^+^/ΔpH = β.

It was experimentally proven that the charged surface of the cartilage resulted from lower friction than that measured at pH 4.2 (pI) for no net charges. Frictional measurements with liposomes and phospholipid bilayers (see Figure 1) supported our proposal that there was low friction within the lamellar-repulsive mechanism [1,2,3]. Low friction is characterized by a small intermediate layer (contact angle ~0^o^), a lamellar slip (Figure 1c) of bilayers, and short-range repulsion. Interfaces and surfaces of articular cartilage that is negatively charged (–PO4^−^) as well as the hyaluronate input, proteoglycans (PGS), a glycoprotein termed lubricin, and the lamellar phospholipid phase [2,3,4], support the concept of a lamellar-repulsive mechanism.

### 2.2. Molecular Dynamics Analysis

In YASARA (Yet Another Scientific Artificial Reality Application, a computer program for molecular visualizing, modeling, and dynamics [29,30,31]), the pH setting only affects the protonation states of the organic molecules and not the solvent composition. PDB files (Protein Data Bank) only provide necessary information about the communication of heteroatoms with slightly low resolution and without hydrogen atoms. Knowledge about the order of covalent bonds is crucial for adding hydrogen atoms or automatically assigning force field parameters. YASARA usually assigns the order of bonds and protonation patterns according to pH 7.0. By changing the pH values, YASARA changes the order of all bonds and then adds the missing hydrogen atoms to match the new pH value.

**Figure 4:**
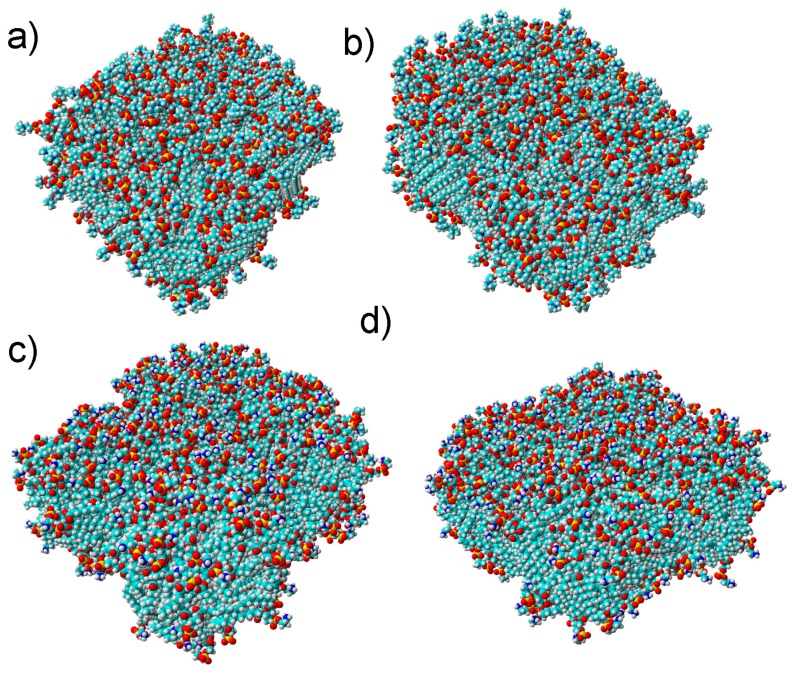
Snapshots from the simulation box. Initial and final structure of the DPPC bilayer (**a**,**b**) and that of the DPPE bilayer (**c**,**d**). Phospholipid atoms are presented by the following colors: oxygen: red, carbon: turquoise, hydrogen: white, phosphorus: yellow.

**Figure 5:**
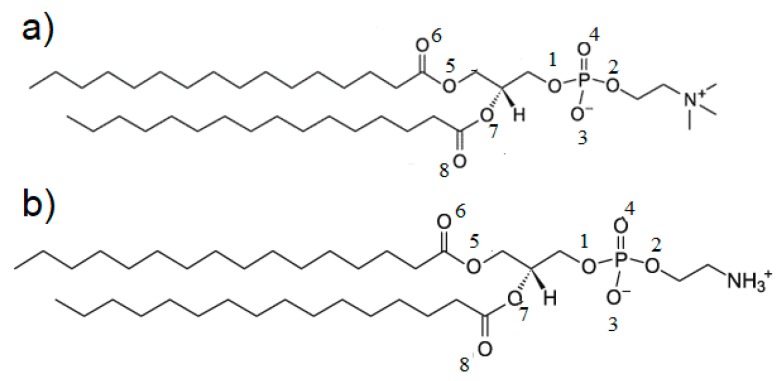
Structures of (**a**) DPPC and (**b**) DPPE with the oxygen atoms numbered.

**Figure 6:**
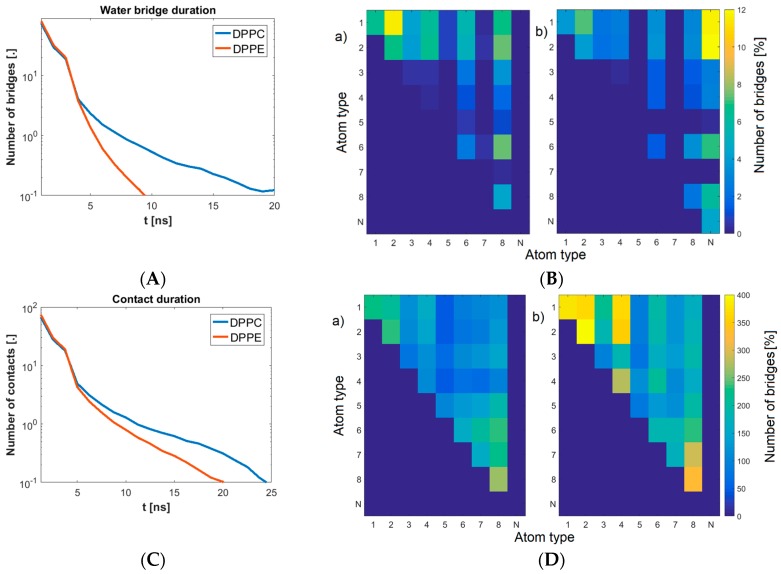
(**A**) Water bridge duration as dependent on membrane type; (**B**) Water bridge map: (**a**) DPPC; (**b**) DPPE. Colors show a relative number of a particular pair as referred to the total number of bridges created; (**C**) Contact between phospholipids in the membrane duration as dependent on membrane type; (**D**) Phospholipids in membrane contact map: (**a**) DPPC; (**b**) DPPE. Colors show a relative number of particular pairs as referred to the total number of bridges created.

Several measures showing the molecular basis of the phenomenon were introduced. Namely, we looked into the water bridge formation between phospholipids (Figure 4 and Figure 5) and the weak H-bond creation between oxygen atoms. By applying these measures, we showed the dynamics of the production of bonds influencing interfacial energy. DPPE created ~150% more water bridges than the DPPC bilayer. However, compared to DPPC, these bridges did not last as long, as shown in Figure 6. There was not much difference between the pH cases in addition to those where pH = 7, where more bridges formed for the DPPC bilayer and fewer for the DPPE. The distribution of these bridges also varied between bilayers.

Moreover, as the water plays an important role in the presented mechanism, we showed an additional map of the water bridges [32]. A water bridge forms when a single water molecule creates H-bonds with both molecules of interest.

Figure 6b shows that while atoms O1 and O2 dominated in the formation of bridges in DPPC, the PE bilayer showed more nitrogen-based bridges. Weak H-bonds between PLs, as seen in Figure 6c,d, occurred more often in the DPPE bilayers (~50% more than DPPC), but did not last as long. These results seem to suggest that each bilayer looks to present a different mechanism of keeping its lipids bonded with each other. DPPC promotes fewer contacts but lasts longer, whereas DPPE shows more connections but does not last as long. Overall, each of these mechanisms seems to yield a similar outcome. 

## 3. Materials and Methods

Phosphatidylcholine (DPPC) and phosphatidylethanolamine (DPPE) of 99% purity were acquired from Sigma-Aldrich (Darmstadt, Germany). With the use of a 20 mg/mL lipid in a n-decane/butanol mixture (3:1) solution, the membrane was created using the Mueller and Rudin method [33].

As the electrolyte solution, the buffer was used in the pH range of 2–12 prepared according to Britton and Robinson [34]. The buffer was prepared by adding 0.2 M sodium hydroxide to 100 mL of the solution (initial pH was 1.81) with the following composition: 0.04 M 80% acetic acid, 0.04 M phosphoric acid, and 0.04 M boric acid (all chemicals from Avantor Performance Materials Poland, Gliwice, Poland).

The proper pH of the buffer was determined depending on the amount of sodium hydroxide added. Buffer pH was changed to, e.g., 3.29 after the addition of 20 cm^3^ NaOH (Avantor Performance Materials Poland, Gliwice, Poland) or to 6.80 if 50 cm^3^ was added. The buffer, as mentioned previously, was used in the experiments because it is widely used in biological studies as a standard buffer due to the wide pH range (2–12) and a composition that does not affect the biological membrane.

### 3.1. Methods of Measuring the Interfacial Tension

Interfacial tension measuring methods have been presented in previous papers [21,35]. The technique that was applied for determining the interfacial tension was established with Young’s and Laplace’s equation (Equation (5)). Thus, the radius of curvature, R, of the convex surface was shaped while a difference of pressure, Δ*p*, was applied to both its sides before being measured. According to Equation (5), the interfacial tension was then calculated [36]:

2*γ* = *R*Δ*p* (5)

### 3.2. Molecular Dynamics Simulations

The AMBER03 all-atom force field [37] was used to study the interactions between the DPPC/DPPE bilayer and water. The DPPC bilayer structure was taken from [38]. Both the DPPC and DPPE bilayers contained 72 lipids; however, to obtain more data, each of them was multiplied by four to obtain a larger structure. The bilayer was simulated for 5 ns to allow the four parts to assemble into one bilayer. After 1000 steps of minimization with a time step of 1 fs, we started the simulations. Bilayer edges were in contact with a simulation box creating an “infinite” bilayer that avoided the collapse of the bilayer into a micelle-like structure. The size of the simulation box was x = 100 Å, y = 90 Å, and z = 90 Å. The TIP3P water model was used [39]. For the isobaric-isothermal (NPT) set, all atomic simulations were carried out under the same conditions: a temperature of 310 K (physiological) and a pH of 7.0, with an aqueous solution of 0.155 M (0.9%) NaCl. The time step was set to 2 fs. Simulations were carried out for 5 ns, and the simulation field was saved every 50 ps. A Berendsen barostat [40] and a thermostat with a relaxation time of 1 fs were used to maintain constant pressure and temperature.

## 4. Conclusions

In this article, we examined the influence of pH on the interfacial tension of lipid membranes and compared it with the friction coefficient of mammalian cartilage/cartilage surfaces results. As the pH was changed, the model membrane created from phosphatidylcholine or phosphatidylethanolamine as well as the friction coefficient in mammalian cartilage showed amphoteric behavior resembling that characterized by the isoelectric point. At pH 7.4, the negatively charged phospholipid bilayers demonstrated a very high buffer effectiveness (β) transmitted by a phospholipid: ΔH^+^/ΔpH = β. This suggests that the lamellar-repulsive hydration mechanism may explain joint lubrication. Therefore, we can conclude that the lamellar slippage of bilayers as well as the short-range repulsion between the surfaces of the negatively charged cartilage surface (–PO4^−^) was the primary determinant of the low frictional properties of the joint, and it was shown that the cartilage surface is a significant factor of the frictional force.

The molecular dynamics simulation results seem to suggest that each bilayer presents a different mechanism by which its lipids maintain bonded with each other. DPPC promotes fewer contacts but lasts longer, whereas DPPE shows more connections but does not last as long. Overall, each of these mechanisms seems to yield a similar outcome.

## Figures and Tables

**Table 1:** The determined parameters for the examined phospholipids: DPPC and DPPE.

Interfacial Tension Values at pI γ_max_ (N/m)	Isoelectric Point (pI) Values	Acid–Base Constant Values [21,24]		Surface Energy [J/mol]
		pK_X_	pK_Y_	
**DPPS [23]**				
2.9 × 10^−3^	3.80	3.36	9.55	1824
**DPPC**				
3.5 × 10^−3^	4.14	2.58	5.69	1800
**DPPE**				
4.0 × 10^−3^	4.20	2.42	5.98	1854

where DPPC = phosphatidylcholine; DPPE = phosphatidylethanolamine; DPPS = phosphatidylserine.
